# Application of Hydrogel in Reconstruction Surgery: Hydrogel/Fat Graft Complex Filler for Volume Reconstruction in Critical Sized Muscle Defects

**DOI:** 10.1155/2016/3459431

**Published:** 2016-06-30

**Authors:** Y. F. Lui, W. Y. Ip

**Affiliations:** Department of Orthopaedics and Traumatology, The University of Hong Kong, Pokfulam, Hong Kong

## Abstract

Autogenic fat graft usually suffers from degeneration and volume shrinkage in volume reconstruction applications. How to maintain graft viability and graft volume is an essential consideration in reconstruction therapies. In this presented investigation, a new fat graft transplantation method was developed aiming to improve long term graft viability and volume reconstruction effect by incorporation of hydrogel. The harvested fat graft is dissociated into small fragments and incorporated into a collagen based hydrogel to form a hydrogel/fat graft complex for volume reconstruction purpose. In vitro results indicate that the collagen based hydrogel can significantly improve the survivability of cells inside isolated graft. In a 6-month investigation on artificial created defect model, this hydrogel/fat graft complex filler has demonstrated the ability of promoting fat pad formation inside the targeted defect area. The newly generated fat pad can cover the whole defect and restore its original dimension in 6-month time point. Compared to simple fat transplantation, this hydrogel/fat graft complex system provides much improvement on long term volume restoration effect against degeneration and volume shrinkage. One notable effect is that there is continuous proliferation of adipose tissue throughout the 6-month period. In summary, the hydrogel/fat graft system presented in this investigation demonstrated a better and more significant effect on volume reconstruction in large sized volume defect than simple fat transplantation.

## 1. Introduction

Replacing lost muscle volume in deep tissue injury, ulcer, and diseases is a common challenge in reconstructive surgeries. The poor regeneration ability of skeletal muscle in critical defects usually results in permanent volume loss. Throughout decades, there are limited achievements in healing facilitation, in terms of both functional and structural recovery, in these defects [1]. Not only the poor regeneration potential but also the complexity of muscle tissue which also involved vascular and neuron networks has hidden the effectiveness of functional regeneration. Compared to the costly regeneration treatments with limited results, simple volume reconstruction offers an applicable and cost-effective alternative for muscle defect treatment among many patients to provide a comparable outcome with current regeneration medicine approach.

In current practice, the autogenic graft commonly employed in soft tissue reconstruction is adipose tissue [2, 3]. Fat graft is a potential candidate for fixing soft tissue defects due to its abundance, availability, and ease to sustain over other soft tissue grafts. Besides its abundance and accessibility in large volume inside human body, adipose tissue is also more resistant to hypoxia which has a good survivability under transplantation. Stem cells inside adipose tissue also provide cell sources for tissue regeneration [4]. Isolated fat graft is able to survive in muscle or subcutaneously for a certain period of time as suggested by some research projects [5, 6]. There are also clinical cases of treating patient suffering from large volume muscle loss or symmetry problem with autogenic fat graft to provide volume reconstruction/filling effect [7, 8]. Autogenic transplantation of adipose tissue, however, is usually subjected to reabsorption which results in severer volume loss after long term implantation [2, 9, 10]. Survival volume of transplanted fat graft lies within a large range between 30 and 80% depending on treatment and location. Another limitation of traditional fat grafting is that there is limited proliferation ability of adipose tissue after transplantation. All these factors limit the effectiveness of fat graft in volume reconstruction therapies. While there are various researches on improving the viability of fat graft during the harvesting process [11, 12], the problem of fat graft survival after transplantation still lacks an effective counter measure.

In total graft transplantation, the transplanted graft is usually subjected to hypoxia which leads to cell apoptosis. Adipocyte apoptosis in graft is usually more severe with the increase in graft volume [13], which results in graft degeneration. This process limits the effect of grafting in large volume defects. In this presented investigation, a new volume reconstruction method based on fat grafting is designed to provide a better volume reconstruction therapy in muscle defects. In the new fat transplantation design, dissociated autogenous fat graft is incorporated into the hydrogel attempting to achieve an improvement in cell viability after isolation. Hydrogel is an effective carrier for autogenic cells during transplantation for enhancing viability [14–16]. The hydrogel carrier is designed to offer media for better diffusion and molecular exchange within the dissociated fat grafts, which enhance the survivability of cells and preserve the progenitors. It can also provide a basement matrix for volume reconstruction and guided tissue formation inside the defect [17]. Primary objective of our hydrogel carrier is to improve the survivability of adipocyte and the progenitors inside transplanted graft in order to reduce degeneration [14] and, in the long run, achieving a long lasting volume filling effect. This hydrogel/fat graft complex was under in vitro and in vivo evaluation on its cell and graft viability preservation ability. Volume reconstruction effect by the hydrogel/fat graft complex was evaluated in an artificially created defect model over a 6-month period and outcomes were compared with simple fat transplantation.

## 2. Material and Experiments

### 2.1. In Vitro Evaluation of Adipose Tissue in Collagen Matrix Gel

A preliminary culture test had been conducted with immortalized cell line. 3T3-L1 preadipocytes obtained from ATCC (ATCC CL-173) were subcultured and expanded under general protocol inside T-75 flask. Prior to cell seeding, culture medium was discarded and the monolayer was rinsed with 1x sterilized phosphate buffer solution. 0.25% w/v trypsin was added to the monolayer and culture under 37°C for 5 minutes until the cell layer was dispersed. Before being incorporated with cells, stock collagen matrix gel was removed from −80°C and thawed inside ice bath for 12 hours until the gel returns to liquid state. Dispersed cells were diluted to 2 × 10^5^ cells/mL by high glucose DMEM before mixing with stock collagen matrix gel in a 1 : 1 ratio. The matrix gel was diluted to final concentration of 50% inside a reaction tube in an ice bath. The mixture was mixed by gentle pipetting to ensure an even distribution. The cell/hydrogel mixture was transferred to a 24-well plate with 1 mL in each well. Culture was performed in a 37° incubator with 0.5% CO_2_ concentration. After gelation for 24 hours, an extra 1 mL of medium was supplemented to the hydrogels. Two phases (hydrogel and culture medium) can be visualized inside the culture well.

Oil red O staining was employed for detection of lipid formation. Cultures were removed from incubator and medium was discarded. The cultures were gently rinsed with sterilized 1x PBS to remove all residue medium. After removing all the PBS by suction, cultures were fixed with 10% buffered formalin at room temperature for 30 minutes. Buffered formalin was discarded after fixation and cultures were rinsed gently with distilled water to remove all residue formalin. 60% isopropanol was added to the culture and allowed to sit for 5 minutes and discarded afterward. Oil red O working solution was added to the cultures and incubated for 5 minutes. Excess stain was removed and cultures were rinsed with tap water after incubation. Cell nuclear cultures were counterstained with hematoxylin. Briefly, hematoxylin was added to the culture until covering all the surfaces and incubated for 1 minute under room temperature. Excess stain was removed and cultures were rinsed gently with tap water after incubation. Cultures were immersed in tap water during imaging under light microscope.

### 2.2. Preparation of Autologous Hydrogel/Fat Graft Complex

Fat graft was harvested from fat pad in the inguinal region of wild type female Sprague Dawley rats after general anesthesia. A 10 mm subcutaneous incision was made on the disinfected region. The inguinal fat was located by blunt dissection and harvested. Skin was closed with 2-O nylon suture by simple interruption. Spray bandage was employed to cover the incision area. The harvested fat pad was cleaned and immersed within saline (0.9% Sodium Chloride Intravenous Infusion) immediately and placed inside an ice bath before transplantation. The harvested fat was immersed in saline and dissociated with surgical scissor to fragments that could be suspended in the saline solution. Dissociated fat graft and fluid were then separated by centrifuge. Upper fluid and blood layer was removed by suction. The dissociated fat graft was then mixed with collagen matrix gel by simple pipetting until an evenly distributed gel is formed. The graft-hydrogel mixture was extracted into a syringe and kept in a 0°C ice bath before application. All experiments were conducted within 30 minutes after harvesting.

### 2.3. TUNEL Staining

APO-BrdU*™* TUNEL Assay kit was purchased from Invitrogen (ref. number A35127). A modified protocol was employed on the assay kit for histological slices staining. Briefly, a DNA-labeling solution was prepared by mixing 10 *μ*L of reaction buffer, 0.75 *μ*L of TdT enzyme, 8.0 *μ*L of BrdUTP, and 31.25 *μ*L of distilled water. The labeling solution was added to the dewaxed, rehydrated sections until the surface was covered and incubated for 60 minutes at 37°C. In the end of the incubation, the sections were rinsed with rinse buffer. All solution was discarded. Antibody staining solution was prepared from mixing 5.0 *μ*L of AlexaFluor 488 dye-labeled anti-BrdU antibody with 95 *μ*L of rinse buffer. Sections were covered with antibody staining solution and incubated for 30 minutes at room temperature in a light proof environment. After incubation, antibody staining solution was discarded and samples were covered with propidium iodide/RNase A staining buffer and further incubated for 30 minutes.

### 2.4. Creation of Critical Sized Muscle Defect and Volume Reconstruction

Wild type female Sprague Dawley rats (Outbred, Albino; Charles River Lab, USA) 9 weeks old were employed in the investigation. All animals were bred and purchased from Laboratory animal unit, The University of Hong Kong (AAALAC accredited 2005). Animals were divided randomly into 3 different groups: investigation group, negative control group (no treatment), and positive control group (simple fat graft transplantation), each group with 9 animals. At surgical operation, all animals were put into general anesthesia by intraperitoneal injection, using Ketamine (90 mg/kg) and Xylazine (10 mg/kg) mixture. Hair over pelvic girdle region was shaved. Skin disinfection was conducted by an iodophor scrub alternated three times with 70% medical ethanol followed by a disinfectant solution. A subcutaneous incision was created on the back of the animal to expose the greater trochanter. A defect of area 10 × 10 mm was created on the greater trochanter by surgical knife down to 3-4 mm deep into the muscle.

**Figure 1:**
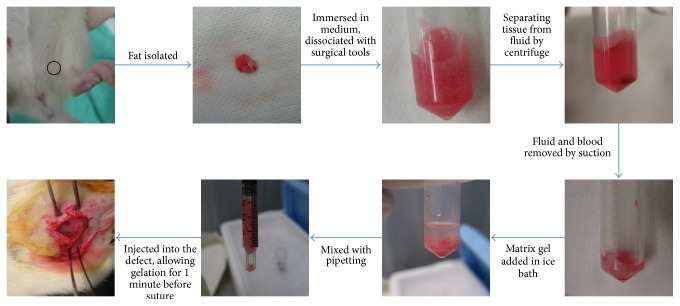
Flowchart of hydrogel/fat graft complex preparation for transplantation into muscle defect.

For the investigation group, the autologous hydrogel/fat graft complex was prepared by the protocol listed above (Figure 1). The complex was injected into the defect until the defect is filled up. The hydrogel was allowed to set and solidify for 5 minutes. Skin was then closed with 2-O nylon suture by simple interruption and covered with spray bandage.

### 2.5. Tissue Processing after Scarification

At respective time points, the animals were sacrificed by an intraperitoneal injection of overdose Pentobarbital (150 mg/kg). Hair over the area of implantation was shaved. An incision was created on the same location as in the pervious operation. The implantation, including any new growth tissue, was harvested with surrounding tissue. Harvested sample was fixed with 10% formalin buffer solution for 4-5 days. After fixation, the samples were rinsed with tap water for 5 minutes before dehydrating in 70%, 90%, and 100% ethanol gradually for 24 hours each, followed by infiltration of xylene for another 24 hours. The samples were infiltrated with Tissue Embedding Medium (Paraplast Plus, Leica) for 24 hours in 60°C with 3 changes under vacuum suction before embedding.

### 2.6. Masson Trichrome Staining

Paraffin section was dewaxed and rehydrated following the standard tissue processing protocol. Dewaxed sections were covered with Weigert's Hematoxylin working solution for 12 minutes, followed by rinsing with tap water for 10 minutes and distilled water for 1 minute. After rinsing, sections were covered with Biebrich Scarlet-Acid Fuchsin working solution and incubated for 1 minute. Excess stain was removed by a quick dip in distilled water. The sections were then incubated with phosphomolybdic-phosphotungstic acid solution for 15 minutes, followed by Aniline Blue working solution for 10 minutes. After removing residue stain on section by a quick dip in distilled water, 1% acetic acid solution was added to the sections and incubated for a further 4 minutes. Stained sections were then dehydrated in 95% and then 100% ethanol, cleaned with xylene, and mounted with DPX.

## 3. Results

### 3.1. Improvement in Survivability of Cells Inside Isolated Graft

**Figure 2:**
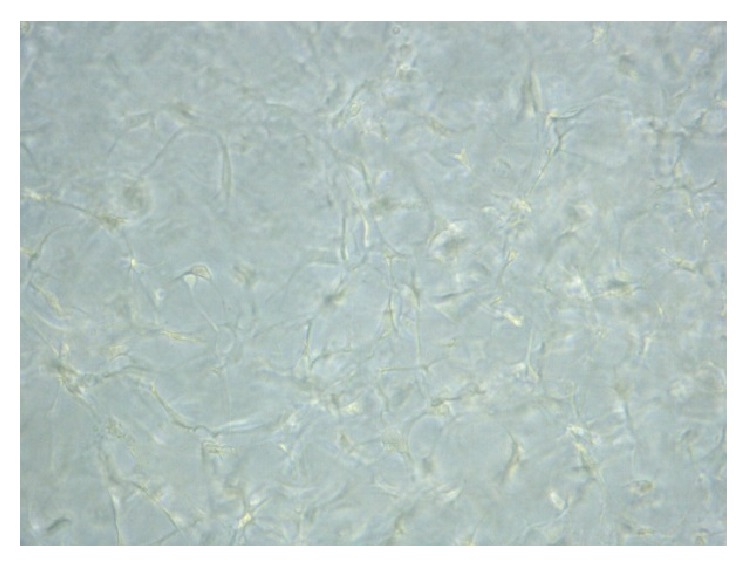
3T3-L1 culture in collagen matrix gel with 1 : 1 dilution, day 7 after seeding. Layers of cells can be observed inside the hydrogel gel matrix. Cell viability and proliferation can be sustained for more than 21 days with constant refreshment of medium surrounding the hydrogel matrix.

**Figure 3:**
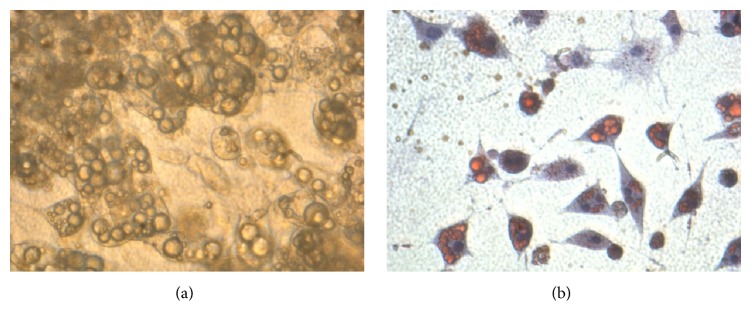
Differentiated adipocyte from 3T3-L1 cells under (a) bright field and (b) oil red R stain. The intracellular oil droplets are stained red and nucleus are stained blue (4x). Collagen gel can support the differentiation of 3T3-L1 cells inside the matrix under influence of growth factors.

Under in vitro culture, preadipocytes were able to survive inside collagen matrix gel under in vitro culturing (24-well plate, 1 mL per well). During the 14-day culture period, depreciation of environment inside the hydrogel could be indicated by the shift in color of phenol red indicator towards acidic ends, but cells continue to survive and proliferate inside the matrix gel which suggests there is still sufficient exchange of molecules between the interior of hydrogel and the surrounding environment. Cell proliferation continues during the culture period. In day 3 to day 7, there was an obvious increase in cell number. Cells were able to proliferate in a three-dimensional manner which nearly occupied the whole volume in the matrix (Figure 2). Multilayer of cells could be found inside the matrix gel. Cells inside collagen matrix gel could be differentiated into lipid containing adipocytes under influence of MDI differentiation medium similar to that in monolayer culture (Figure 3). First appearance of lipid droplets inside cells could be observed on around day 10 after incubation, which was delayed by about 3-4 days when compared to monolayer culture. After 14 days, a considerable amount of cells could be found containing lipid droplets within the cytoplasm.

**Figure 4:**
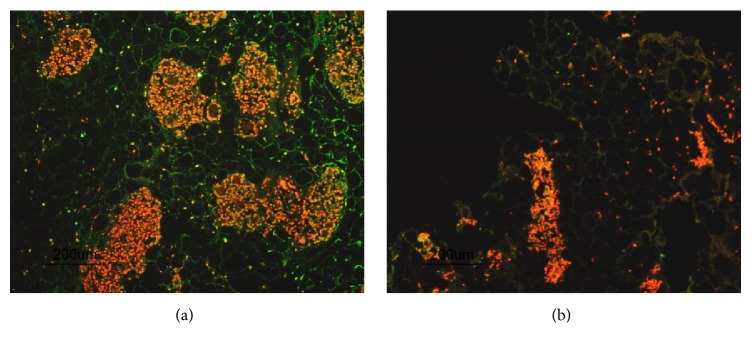
Comparison between isolated fat grafts under in vitro culture before and after hydrogel incorporation ((a) normal fat graft and (b) hydrogel/fat graft complex). Apoptosis cells are marked with green and living cells are marked with red fluorescent. An improvement in cell viability inside the graft can be indicated by the reduced amount of green light and observable red fluorescent signal from living cells.

Adipose tissue structure of harvested fat graft could usually be preserved for the first few days under in vitro culture after isolation. TUNEL stain however reveals that large amounts of cells were undergoing apoptosis under in vitro culture, indicated by DNA strand breaks, after 3 days from isolation despite of their intact extracellular matrix (Figure 4). Remaining living cells were closely packed from each other and concentrated in separated colonies outside the adipose tissue matrix. There was an overall low cell survival ratio inside the isolated graft.

Cell survivability inside the isolated fat graft could be significantly improved after dissociation and incorporated into hydrogel. This hydrogel/fat graft complex was prepared identically to the protocol employed in later animal study. While sectioning indicates some loss in adipose tissue matrix caused by the dissociation, there was in general a reduction in apoptotic cells inside the adipose graft. A larger number of living cells could be located inside the adipose tissue matrix (Figure 4). With the same volume of harvested tissue, hydrogel/fat graft complex had shown a significant improvement in cell viability over traditional fat grafting under in vitro culture.

### 3.2. Volume Reconstruction in Muscle Defects

The hydrogel/fat graft complex was injected directly into artificially created defects. To prevent leakage of hydrogel from the area usually 5–7 minutes was allowed for gelation before closing the incision. Instead of a homogenous solution, the graft-hydrogel complex represents a paste-like filler with dissociated graft saturated inside a collagen matrix. No leakage of hydrogel complex could be observed after closure of surgical site. All animals survive the surgery and showed no negative effect caused by the transplantation. There were swallowing and blood infiltration into the surgical site in the first 1 to 2 days after the operation. No chronic inflammation had been developed in the observation period. All animals were able to restore normal behavior within 2 weeks. The walking pattern and locomotion were not affected by the surgery.

Signs of the healing progress in the first month between groups treated with the hydrogel complex and simple fat graft transplantation was comparable to each other. In both groups, the transplanted graft changed to brownish from original white color. There was formation of fibrous tissue in the surgical site. Compared to direct autogenic graft transplantation, the hydrogel complex offers a more even filling effect in the muscle defect: The hydrogel based filler covers the defect evenly rather than at a single concentrated spot like simple fat grafts (Figure 6). There was a slight reduction in total hydrogel/fat graft volume in early time points, which reexposes the defect to visible level.

**Figure 5:**
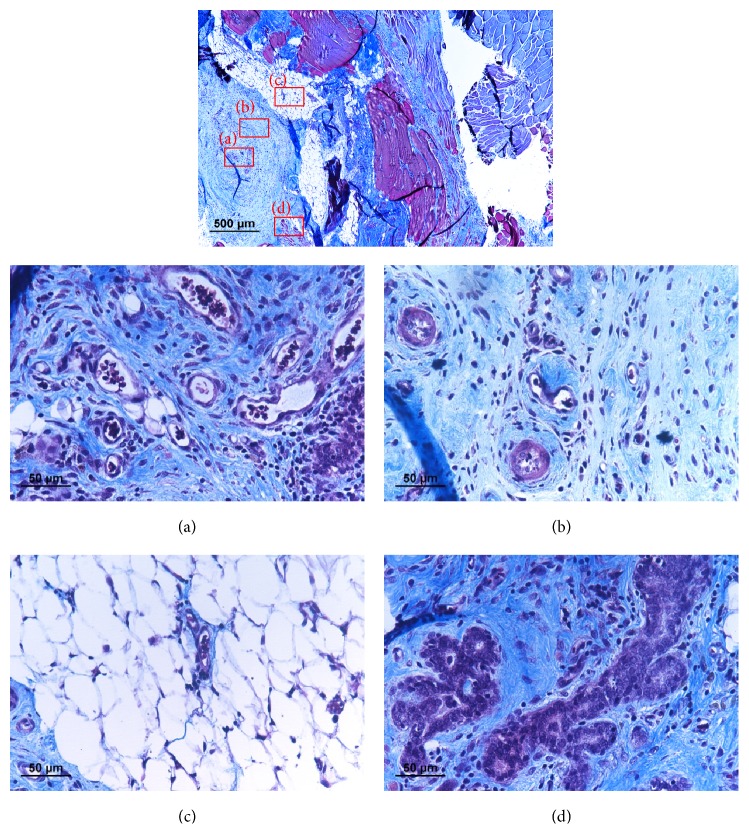
Tissue section collected inside the muscle defect after being treated with hydrogel/fat graft complex after 1 month healing under histological stain (Masson Trichrome). (a) Blood vessel can be found in the center of the area (40x). (b) Formation of collagen rich fibrous tissue (40x). (c) The remaining adipose tissue represents only a small volume inside the defect (40x). (d) Degenerated adipose tissue similar to those found in simple fat transplantation can be found (40x).

**Figure 6:**
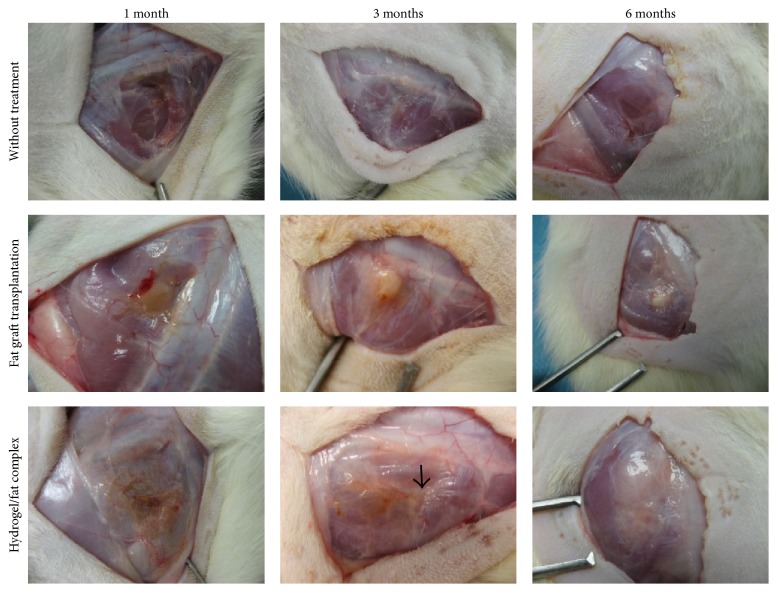
Artificial created muscle defect with different treatment in 6 months' healing period. Comparing defect treated with hydrogel/fat graft complex and simple fat graft transplantation, a more even and smooth fat pad can be observed at the 6-month time point. First trace of new fat pad development could be observed in 3-month time point (black arrow). There is a continuous development of fat pad throughout the observation period with the hydrogel/gat graft complex group. Tissue volume is fully restored by the fat pad after 6 months.

Consider the whole defect region, larger amount of newly formed tissue could be identified on animals treated with hydrogel/fat graft complex compared to other experimental groups (Figure 6). Histological study reveals that only small amount of adipose tissue could preserve its structure after one month within the hydrogel/fat graft complex after transplantation (Figure 5). Majority of defect volume was filled up with collagen rich fibrous tissue. There was also extensive angiogenesis inside the treated area.

**Figure 7:**
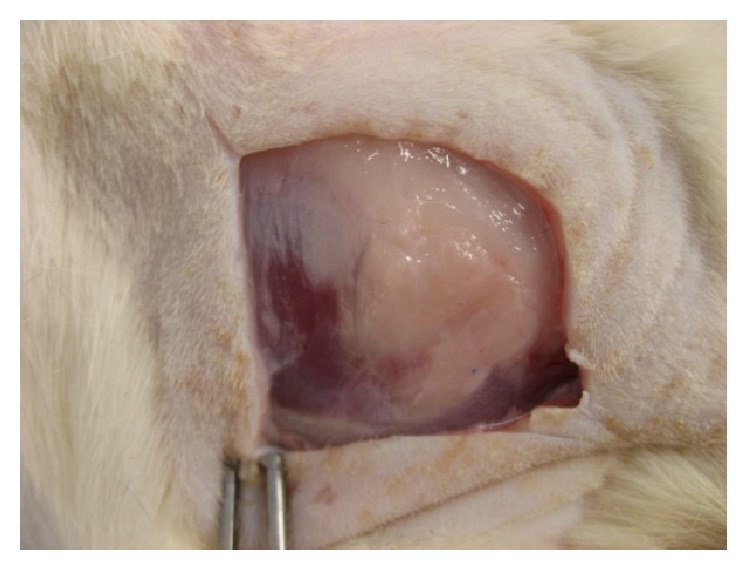
Muscle defect treated with hydrogel/fat graft complex after 1-year healing period. The fat pad continuous to survive with no traces of degeneration or reduction in volume.

Feasible effect induced by the hydrogel/fat graft complex began to appear after 3 months from operation. Different from simple fat graft transplantation where there was almost no proliferation or regeneration of adipose tissue, formation of new fat began to appear on the defect site at the 3-month time point on the animals group treated with hydrogel/fat graft complex (Figure 6). Majority of original transplanted graft could still be located inside the defect volume indicated by the brownish color. On the other hand, newly formed tissue in hydrogel complex system began to provide a much more even volume filling effect than simple fat graft transplantation. These newly formed adipose tissues continue to develop inside the defect volume in 6-month time period. Fat tissue began to fill up the whole volume evenly inside the muscle defect in the group treated with hydrogel/fat graft complex (Figure 6). In about 75% of subjects, large fat pads completely filled up the whole defect area, fully restoring the original tissue volume. This observation occurs mostly after 6 months of the healing. There were differences in both tissue volume and nature when compared to simple fat transplantation at this respective time point. One-year follow-up investigation had revealed that these large fat pads are able to survive and could retain their volume, free from extensive degeneration and fibrosis. In some cases, there was even a further development in fat pad volume during the 1-year period (Figure 7).

**Figure 8:**
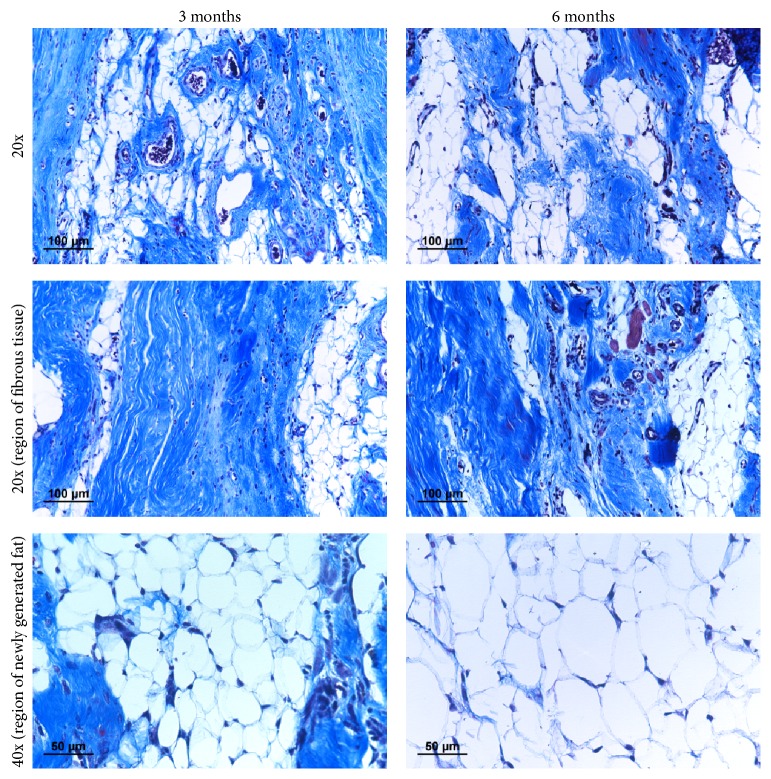
Tissue healing process in muscle introduced with hydrogel/fat graft complex in 3-month and 6-month time points, with histological study preformed with Masson Trichrome stain. Mature collagen fibre exists inside the area, and there is a gradual increase in amount of fat tissue. Newly formed capillaries (RBC stained in black) can also be found inside the area.

**Figure 9:**
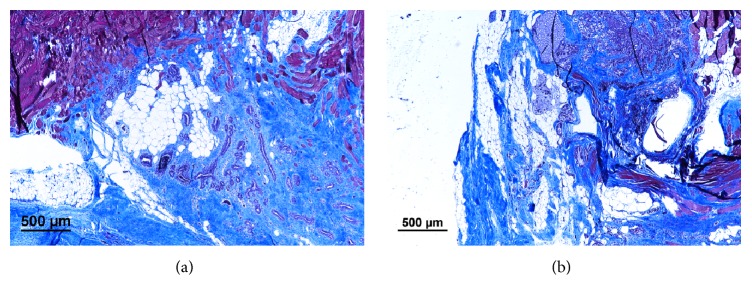
Tissue healing process in muscle introduced with (a) simple fat graft and (b) hydrogel/fat graft complex in 6-month time point. (4x) Most of the adipose tissue has degenerated into fibrous tissue in simple fat graft transplantation, while subjects treated with hydrogel/fat graft complex show traces of adipose tissue developing in between fibrous tissue. It is noticeable that there is a more even distribution of adipose tissue inside the collagen based fibrous tissue for subjects introduced with hydrogel/fat graft complex.

Histological study reveals that there was continuous change in tissue type inside the hydrogel complex during the 6-month investigation period. The amount of neutrophils had greatly reduced after the first month. In certain area, the fibrous matrix was gradually replaced by adipose tissue and a large fat pad composed of mostly adipose tissue was formed after 6 months. During the transformation, collagen matrix still dominates most volume inside the defect. Organized fibrous tissue could be found in 3-month time period, running across the newly regenerating fat (Figure 8). Compared to simple fat transplantation where majority of adipose tissue was transformed to mature fibrous tissue, the hydrogel/fat graft complex offers a significant improvement in maintaining or restoring viability of transplanted fat graft (Figure 9).

## 4. Discussion

In reconstructive surgeries, how to maintain the defined volume of reconstructed tissue is an essential consideration. Current soft tissue reconstruction methods with fat graft are usually subjected to volume reduction during the healing period, sometimes up to 70% of original volume. These reductions in graft volume are caused by the natural remodeling and the degeneration process inside human body. How to improve the viability of graft under long term transplantation is therefore an important consideration for volume reconstruction [18]. In our investigation, collagen matrix gel was employed as a carrier for the fat grafts in order to provide a solution to these problems. Compared to the natural adipose tissue matrix, hydrogel allows easier molecular penetration to both water and biomolecules. This facilitates diffusion of molecules to the grafts which can improve the survivability of cells [19]. The hydrogel also acts as a reservoir for nutrients during early transplantation. In addition, hydrogel gel also offers a suitable matrix for cell and new tissue proliferation. The objective of hydrogel in our investigation is to improve the survivability of progenitor cells inside the transplanted graft to ensure long term viability and development. As shown in our in vitro evaluation, a higher cell viability ratio can be achieved with dissociated fat graft saturated in hydrogel compared to total graft transplantation.

The introduction of hydrogel cannot eliminate the degeneration of fat graft during initial stage of transplantation. There is a loss in adipose tissue structure and a reduction in graft volume like tradition fat grafting. Histological study shows extensive fibrous tissue formation inside the defect area, where the wound is filled by collagen rich fibrosis tissue. It is uncertain whether these collagen fibres are newly deposited matrix from host cells or the residue of the collagen matrix gel. Limited traces of adipose tissue can be found inside the site at the one-month time point. The dissociation of fat graft results in the increase in surface area to volume ratio which leads to faster degeneration of structure compared to simple autogenic graft transplant. Early response after transplantation, signature by volume shrinkage and structural degeneration, of hydrogel-graft complex is comparable to simple fat grafting.

While the early response is similar between the two treatments, advantage of the hydrogel incorporation can be demonstrated in long term healing. Our observation suggests after the early degradation phase that there is a continuous increase in the amount of adipose tissue inside the muscle defect site after the introduction of the hydrogel/fat graft complex filler. This process can take place without artificial introduction of growth factors. These newly generated fat pads located both inside and on the surface of the volume defect. Six months after the operation, a fat pad is composed of a mixture of adipose and fibrous tissue forming a large tissue bulk covering the whole defect area. Our one-year observation suggests that these fat pads can maintain its volume throughout the period. Within the same period, there is replacing of fibrous tissue with fat in the defect area. The introduction of hydrogel can ensure a higher survivability ratio of cells inside the dissociated fat graft, which possibly can encourage the new fat formation. Progenitors can also be preserved in a better manner which can differentiate into fat cells for further new tissue formation [20]. Despite the more extensive loss in matrix structure in early stage, the hydrogel complex system likely allows a sustainable development and growth of adipose tissue in the implantation site.

Autogenic graft was chosen over autogenic cells in volume reconstruction for large volume muscle defects in our project. Isolated autogenic cells offer a higher effective progenitor cells concentration than autograft which in theory can provide a better tissue regeneration effect under influence of growth factors. Bulk adipose tissue is able to regenerate from isolated adipose-derived stem cells as demonstrated in various research projects and is sometimes considered more effective than simple graft transplantation [4, 21, 22]. In clinical situation, however, it is often impossible to obtain enough autologous progenitor cells in effective dosage within a short period of time. The complicated and time consuming process for cell isolation and expansion makes the treatment unfavorable for actual clinical application in many cases. Isolated cells are also more prone to leakage from the targeted area. In these circumstances, autogenic graft offers a more promising effect in volume reconstruction. Not only can natural signaling molecules be preserved inside the extracellular matrix, but also they offer an extra supportive structure for volume filling and localization purpose. In the above results, we have demonstrated that hydrogel/fat graft complex can achieve good cell transportation effect for long term tissue regeneration in certain application without the need of isolation and expansion.

The results from the animal test are very encouraging. It is possible to create a thick and structural stable fat pad with desired shape and dimension in targeted location to achieve a volume reconstruction effect. Formation of thick fat pad is successful in about 75% of the animals we employed, while the remaining 25% also showed traces of fat pad formation inside the hydrogel introduced area. Most of the fat pad measures a larger volume than the autograft originally harvested. The newly formed fat pad can maintain its viability in our one-year observation without degeneration and shrinkage which offers a much appealing result than simple fat transplantation. Dissection reveals that the formation of fat pad is only restricted to the area where the hydrogel/fat graft complex appears. Some of this newly formed fat is found subcutaneously, which is highly possible to be originated from the leakage of hydrogel filler into subcutaneous space from the defect site during surgical operation. There is however no accurate statistical data about this observation in current status.

## Additional Points

In summary, great success in long term volume reconstruction has been achieved by employing collagen matrix gel incorporated fat graft. Different from simple fat graft transplantation and artificial fillers, the hydrogel complex cannot provide an immediate effect in volume reconstruction. Long term investigation however reveals that the hydrogel complex is able to facilitate formation of fat pad inside the targeted area. A thick layer of adipose tissue can be developed inside the muscle defect where the hydrogel complex was introduced after 6 months. Different from simple transplantation, these newly generated adipose tissues can offer a healthy fat pad with normal adipose tissue anatomy free from fibrosis. Long term investigation reveals that the fat graft can be maintained and continues to develop for over 1 year. The fat pads offer a nearly complete restoration of volume inside the muscle defect of our animal model. It is notable that the newly generated fat pad also excesses the original volume of the harvested fat graft. All the evidence in our investigation suggests that this hydrogel/fat graft complex system can offer a new way in fat transplantation and volume reconstruction.
